# Fifteen-month-old infants use velocity information to predict others’ action targets

**DOI:** 10.3389/fpsyg.2015.01092

**Published:** 2015-08-04

**Authors:** Janny C. Stapel, Sabine Hunnius, Harold Bekkering

**Affiliations:** Donders Institute for Brain, Cognition and Behaviour, Radboud University NijmegenNijmegen, Netherlands

**Keywords:** action prediction, infancy, speed-accuracy trade-off, motor system, predictive eye-movements

## Abstract

In a world full of objects, predicting which object a person is going to grasp is not easy for an onlooker. Among other cues, the characteristics of a reaching movement might be informative for predicting its target, as approach movements are slower when more accuracy is required. The current study examined whether observers can predict the target of an action based on the movement velocity while the action is still unfolding, and if so, whether these predictions are likely the result of motor simulation. We investigated the role of motor processes for velocity-based predictions by studying participants who based on their age differed in motor experience with the task at hand, namely reaching. To that end, 9-, 12-, and 15-month-old infants and a group of adults participated in an eye-tracking experiment which assessed action prediction accuracy. Participants observed a hand repeatedly moving toward and pressing a button on a panel, one of which was small, the other one large. The velocity of the reaching hand was the central cue for predicting which button would be the target of the observed action as the velocity was lower when reaching for the small compared to the large button. Adults and 15-month-old infants made more frequent visual anticipations to the close button when it was the target than when it was not and were thus able to use the information in the speed of the approach movement for the prediction of the action target. The 9- and 12-month-olds, however, did not display this difference. After the eye-tracking experiment, infants’ ability to aim for and press buttons of different sizes was evaluated. Results showed that the 15-month-olds were more proficient than the 9- and 12-month-olds in performing the reaching actions. The developmental time line of velocity-based action predictions thus corresponds to the development of performing that motor act yourself. Taken together, these data suggest that motor simulation may underlie velocity-based predictions.

## Introduction

Predicting others’ actions is crucial for social interactions to run smoothly (Bekkering et al., 2009; Sebanz and Knoblich, 2009). Anticipating which goal object an action partner will grasp, however, is complicated in a world full of objects. How do observers predict which object another person is reaching for? And how does the ability to predict others’ actions develop early in life? Motor theories of action perception suggest that the motor system is used to predict others’ actions the same way it is used to predict the outcomes of one’s own motor acts (Wolpert et al., 2003; Oztop et al., 2005; Prinz, 2006; Kilner et al., 2007a). In accordance with this notion, a large body of literature shows that the motor system is not only active during action execution but also during the observation of others’ actions (Rizzolatti et al., 1996; Hari et al., 1998; Cattaneo et al., 2007), suggesting that similar processes are at work during observation and execution. Consequently, laws governing action production can be expected to also affect action perception. One of these laws is Fitts’s law (Fitts, 1954), which describes that actions directed at small targets require more time to perform. Recent empirical findings illustrate that observers have expectations about the speed of an observed movement depending on the size of the target (Grosjean et al., 2007) and that these expectations follow Fitts’s law. However, it is yet unclear whether this law is used to predict ongoing observed actions. If so, this would allow people to predict the target of a partner’s actions when many potential targets are present. The first question of the current research was whether observers indeed can use the velocity of an action to *predict* whether an action is directed at a small or large object. The key advantage of action prediction over mere processing of completed actions is that prediction allows for smooth and timely social interaction (Bekkering et al., 2009; Sebanz and Knoblich, 2009). A second aim of the study was to investigate which mechanism underlies velocity-based predictions by taking a developmental approach. Given the large body of literature suggesting that the motor system is involved in action prediction (Wolpert et al., 2003; Oztop et al., 2005; Prinz, 2006; Kilner et al., 2007a) and prior empirical evidence that Fitts’s law affects action observation (Grosjean et al., 2007; Eskenazi et al., 2009), it is plausible that motor simulations bring about velocity-based predictions. As a second question we therefore examined whether motor development goes hand in hand with the development of velocity-based predictions, by employing a cross-sectional design.

When acquiring a novel motor skill, the actor builds associations between the motor commands utilized and the effects of these motor commands as experienced via the sensory modalities (Miall and Wolpert, 1996; Kawato, 1999). At first, gaze is directed at the effectors (hands, fingers, feet) to monitor the results of the new motor commands (White et al., 1964; Sailer et al., 2005). With action proficiency, gaze will no longer be directed at the effectors, but at the target of the action (Sailer et al., 2005) and hence reveals the target of the ongoing action. Based on associations formed during the acquisition phase, a forward model of the action can be constructed, which allows the actor to predict the sensory consequences of an intended action ahead of time (Wolpert, 1997). The forward model becomes more fine-grained with increasing motor experience. In this way, motor experience leads to a precise forward model of the action and to precise predictions of future sensory states.

Motor theories of action perception assume that similar processes are active during action perception as during action production (e.g., Oztop et al., 2005). Numerous studies have demonstrated that brain areas responsible for action production are activated during action perception as well (Hari et al., 1998; Buccino et al., 2001; Cattaneo et al., 2010). The observers’ motor system of both adults (Calvo-Merino et al., 2005, 2006) and infants (van Elk et al., 2008) appears to be more activated during observation of acts that are firmly established in the observers’ motor repertoire compared to more novel motor acts. On a behavioral level, goal-directed eye movements have been shown to be predictive and follow the same time course for action execution and action observation (Flanagan and Johansson, 2003), and blocking the motor system by means of Transcranial Magnetic Stimulation (TMS) disrupts these predictive eye movements (Elsner et al., 2013). Eye-tracking studies investigating the development of action prediction indicate that motor experience is crucial for predicting these actions in others (Falck-Ytter et al., 2006; Kanakogi and Itakura, 2011; Ambrosini et al., 2013; Stapel et al., submitted). Participants with difficulties in planning their own action sequences, namely children with autism, show also less indications that they predict others’ actions, whereas typically developing children anticipate their own next action, and a similar predictive muscle activation is found when they observe the same action in others (Cattaneo et al., 2007; Fabbri-Destro et al., 2009). Based on these findings, it is therefore likely to assume that velocity-based predictions become more accurate as a consequence of motor development.

In action performance, speed depends on the accuracy required for successful completion of the action. That is, the more accurate one has to be, the slower the movements become. Fitts (1954) formalized and quantified this relation based on data he collected, and the relation he found was shown to hold for many movements (see for an overview Plamondon and Alimi, 1997). Fitts’s law states that the time needed to move between two targets is based on the distance between the targets and the width of the target (Fitts, 1954). Hence, average velocity can be higher between large compared to small target objects, and bridging small distances can be done quicker than bridging large distances. For example, reaching and grasping a small object requires more accuracy, and has been shown to take more time (Bootsma et al., 1994; Zaal and Thelen, 2005).

Empirical research shows that in adults, not only action production follows Fitts’s law; also action perception is influenced by it. For instance, adults were capable of dissociating whether an observed, artificial reaching movement was physically possible or impossible in reality given the average velocity, adhering in their judgments to Fitts’s law (Grosjean et al., 2007). Also, a neurophysiological patient violating Fitts’s law in his action production by not adjusting movement speed for smaller targets displayed similar violations in action perception (Eskenazi et al., 2009). This indicates that determining whether observed actions have an appropriate velocity might be grounded in the action production capabilities of the observer. Presumably, the neural motor system is recruited during action perception to simulate the observed action. These simulations during action observation may enable the observer to predict future states of the action (cf. Wilson and Knoblich, 2005). An fMRI study by Eskenazi et al. (2011) revealed that activity in motor areas of the brain during the observation of movements was related to the difficulty of performing these movements as formalized in Fitts’s law. In sum, the speed-accuracy trade-off not only constrains action production, it also affects action observation, and these constraints influence activity in motor cortical areas of the brain during observation and execution. The speed-accuracy trade-off has primarily been studied in adults; little is known about the development of the perception of actions that differ as a consequence the speed-accuracy trade-off. The current study takes a novel approach by investigating the mechanisms underlying processing of the speed-accuracy trade-off from a developmental perspective.

The study was set out to investigate whether observers not only use the speed-accuracy trade-off to dissociate possible from impossible actions, but whether they also use this principle to predict the targets of actions they observe. Furthermore, if the motor system generates target predictions based on the velocity of the observed movements, then these predictions can only be made by observers capable of performing the observed action herself, because before skill acquisition, the observer most probably lacks the necessary forward model to predict the action outcome. We therefore adopted a developmental approach: 9-, 12-, and 15-month-old infants participated together with adults in an eye-tracking experiment during which they observed an actor moving her hand toward and pressing a large or a small button. In all stimulus videos, there were two buttons, a large and a small one, at the end of a table. A hand started moving from the side of the table and to the other to press either the large or the small button. Natural movements were used in the stimuli, resulting in slower movements toward the small button than to the large button. If participants made more correct visual anticipations than incorrect anticipations, then that would form an indication that the observers used the velocity of the hand to predict whether a specific button would be pressed or not. We hypothesized the ability to predict others’ aiming and pressing actions to develop in parallel with their own ability to accurately aim their hand and finger at a small target in order to press it. Pressing a small button requires the use of the index finger independently from the other fingers. This ability is also needed to grasp small objects with the pincer grasp. At 8 months of age, typically half of the infants is capable of performing the pincer grasp (van der Meulen et al., 2002). Infants begin to use the pincer grasp more frequently and more precisely as they get older. These developmental changes occur mainly until 15 months of age, as the use of the power (whole hand) grip decreases (Butterworth et al., 1997). Young infants might thus be able to successfully aim with their hand for a large button, but they might base their movements on a relatively inaccurate forward model, which prevents them from smoothly reaching for and pressing a small button. Having a coarse-grained forward model might necessitate them to make corrections in their movements if they would try to aim for and press a small button. At the same time, this coarse-grained forward model might not allow them to make accurate predictions of other’s actions. To further clarify the role of motor expertise for velocity-based action prediction, the infant groups were tested for their ability to aim at a small button. This allowed us to disentangle whether potential developments in predicting targets based on speed arise specifically from the development of the motor skill at hand or rather reflect other age-related changes.

## Materials and Methods

In the following section, we report the way sample size was determined, all data exclusions, all manipulations, and all measures of interest for the study.

### Participants

Due to the innovative nature of the study, it was impossible to perform a reliable effect size estimation based on previous studies, rendering the study exploratory. We aimed to gather data of at least 24 infants per age group. As adults provide more stable gaze data and are better capable of attending for longer durations, we aimed for testing 18 adults. In this type of study, there are two forms of drop out: immediate drop-outs due to insufficient gaze calibration or infant distress and failure to collect enough gaze data. The first form can be noticed during testing and hence this type of drop out can immediately be replaced. The second form can only be discovered during the analyses. Drop-outs that occurred during the analyses were not replaced. For that reason, sample sizes vary slightly between the groups.

Twenty-seven infants (eight girls) with a mean age of 8.8 months (SD = 0.3), 28 infants (16 girls) with a mean age of 12.2 months (SD = 0.3), and 28 infants (11 girls) with a mean age of 15.0 months (SD = 0.2) participated in the study. Furthermore, 18 adults (12 women, mean age = 24.9 years, SD = 5.2) took part in a longer version of the experiment. Eight additional infants (three 9-month-olds, five 12-month-olds) and one additional adult were tested but excluded from the analyses because they did not meet the eye-tracking calibration criteria (seven infants) or because they produced an insufficient amount of gaze data (gaze data for only three or less trials: one infant, one adult). The production task of 12 infants (six 9-month-olds and six 12-month-olds) could not be analyzed as it turned out to be difficult to videotape the action execution task from an angle at which both the infant, the infant’s hand and the device was visible at all times. In nine cases, (part of) the action was not visible in the video, rendering it impossible to code the behavior later on. In three other cases, the action was not recorded due to experimenter error. All infant groups were recruited via the Baby Research Center in Nijmegen. The adults were recruited via a participant database of Radboud University Nijmegen. Written informed consent of the participants or the participants’ parents was obtained prior to participation. Participation in the study was rewarded with a small gift (an infant book or 10 Euros for the participating infants, 5-Euro-gift vouchers or credit points for the adults). The study was approved by the ethical committee of behavioral science at the Faculty of Social Sciences in Nijmegen (approval number ECG2012-1301-006 for the infant participants and approval number ECG2012-0910-058 for the adult participants), and was conducted in conformity to the ethical standards of (developmental) psychology.

### Stimuli

**FIGURE 1:**
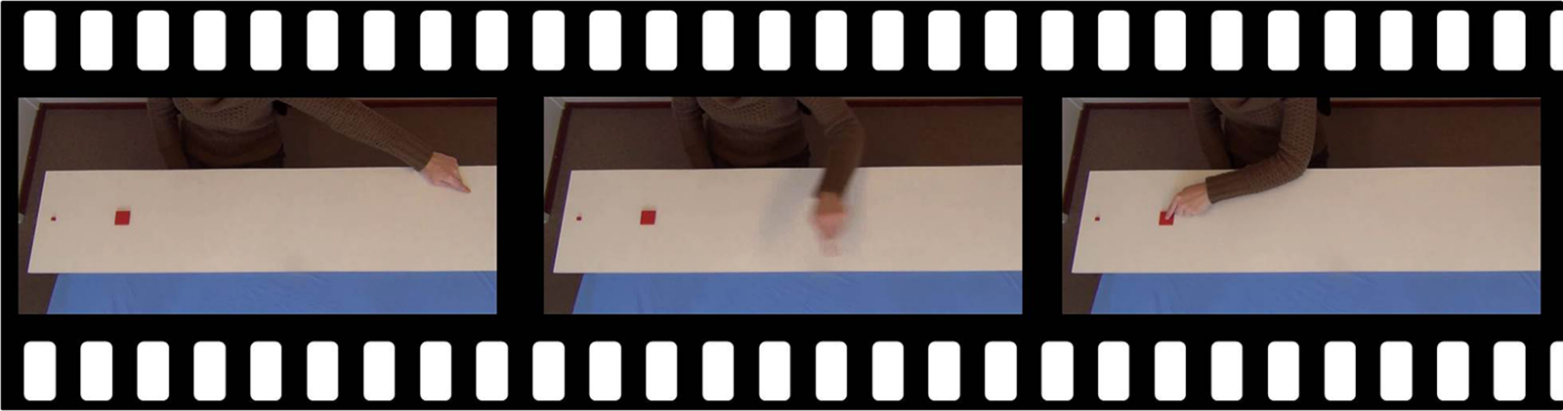
**Example frames from a stimulus video with the small button near the edge of the table and the hand moving to the large button**.

Four different short video clips (duration: 3.1–3.6 s) were used as stimulus material. The videos showed a table with a large (4 by 4 cm) and a small (1 by 1 cm) button on one side (see **Figure 1**). Velocity of natural movements directly impacts the height of the movement trajectory: slow movements allow for a stronger curvature than fast movements (Lacquaniti et al., 1983). To minimize potential effects of movement trajectory, the actions were filmed from a near top view. An actor was sitting behind the table. One of the buttons was relatively near the edge of the table, and the other one was a bit further away from the edge toward the middle (center-to-center distance between the buttons was 20 cm). In half of the videos, the small button was the one closer to the edge of the table, whereas it was the large button in the other half of the videos. The stimulus videos started with a still frame in which the actor’s hand was shown on the far side of the table. To create a balanced stimulus set, also horizontally flipped versions of the videos were made by means of editing the original video material in VirtualDub (www.virtualdub.org). After 1 s, the hand started moving toward the buttons, and the action ended with the hand pressing one of the buttons with the index finger. The video ended with 1 s of still frame of the hand in its end position with the index finger pressing the button. This could be either a small and far, small and close, large and far, or a large and close button. Natural reaching movements were used because biological motion processing is thought to be disrupted by artificial compared to natural movements (Servos et al., 2002; Kilner et al., 2007b). The actress was instructed to fixate at the target throughout the trial and to direct her head to a fixed point in space on a line intersecting the midpoint between the two buttons, thereby avoiding potential cues of shoulder direction to influence the predictions of observers. The index finger was already stretched out during the start of the movie, such that during movement the fingers did not move with respect to the hand. As expected based on Fitts’s law, movements toward the small buttons took more time than movements toward the large buttons (300 ms difference), and pressing the distal button required more time than pressing the proximal button (20 ms difference). The resulting average velocity of the hand until it reached the area of interest around the closest button ranged between 988 and 1522 px/s. The average velocity of the hand was 1222 px/s moving toward the large close button, 1522 px/s toward the large far button, 988 px/s toward the small close button, and 1240 px/s toward the small far button. Hence, the average velocity of the natural movements was manipulated by means of manipulating the size of the target button as well as the distance to the target button.

**FIGURE 2:**
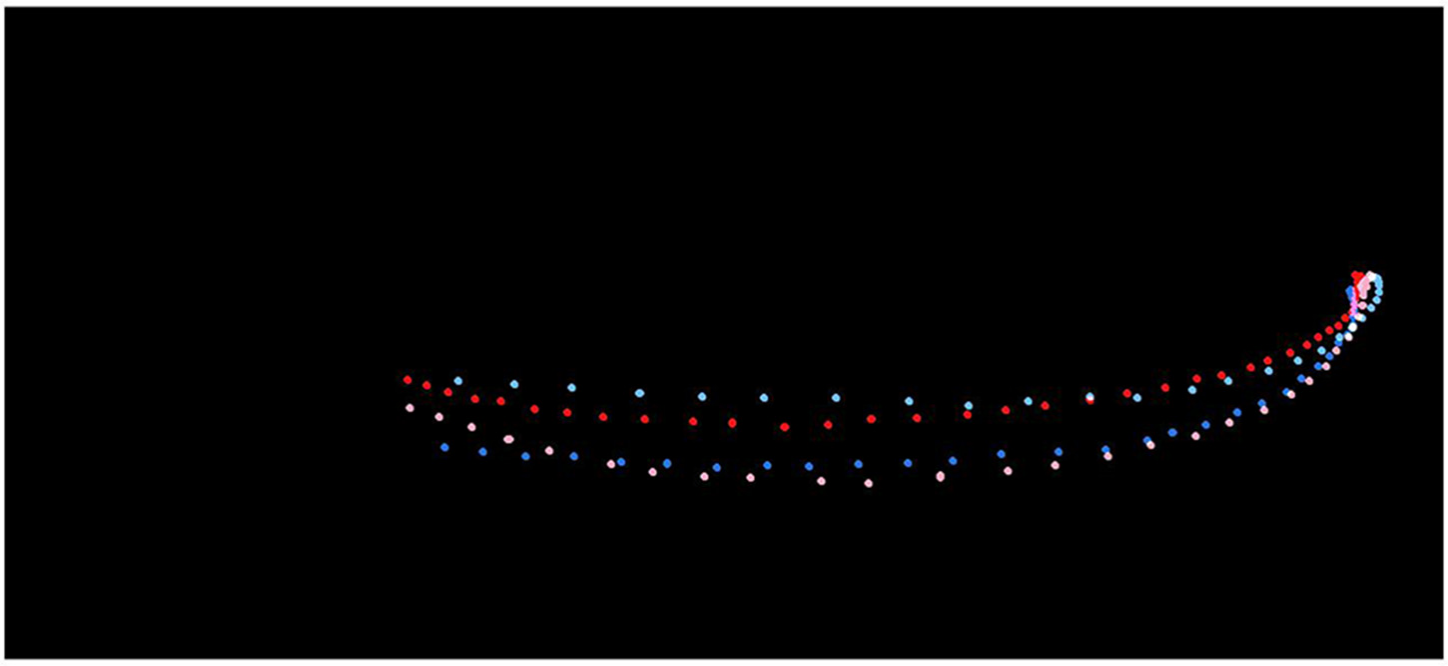
**Illustration of the motion paths used in the stimuli**. The pink dots represent the motion path of the action toward the large close target, the red dots represent the action toward the small close target, the light blue represents the action targeted at the large far button, and the dark blue represents the action targeted at the small far button.

The motion paths of the actions have been visualized to give more insight into the variability between the stimuli, see **Figure 2**. The image was constructed in the following way: (1) The frames from the period of interest per stimulus video were saved as bitmaps, (2) The location of the tip of the index finger was marked per frame with a colored dot. (3) The images were read in frame by frame using Matlab R2014b (MathWorks Inc.) and the locations of the colored dots were stored into a matrix per video, (4). The four matrices were added and plotted. The figure illustrates that natural reaching movements indeed are variable, but no clear pattern is visible revealing that the one type of paths leads to the far and another type to the close button: the blue paths are not very similar to each other, nor are the red paths. The red paths continue further to the left, which illustrates that these actions decelerated at this point, whereas the other two actions continue on full speed at this point.

### Button Press Device

**FIGURE 3:**
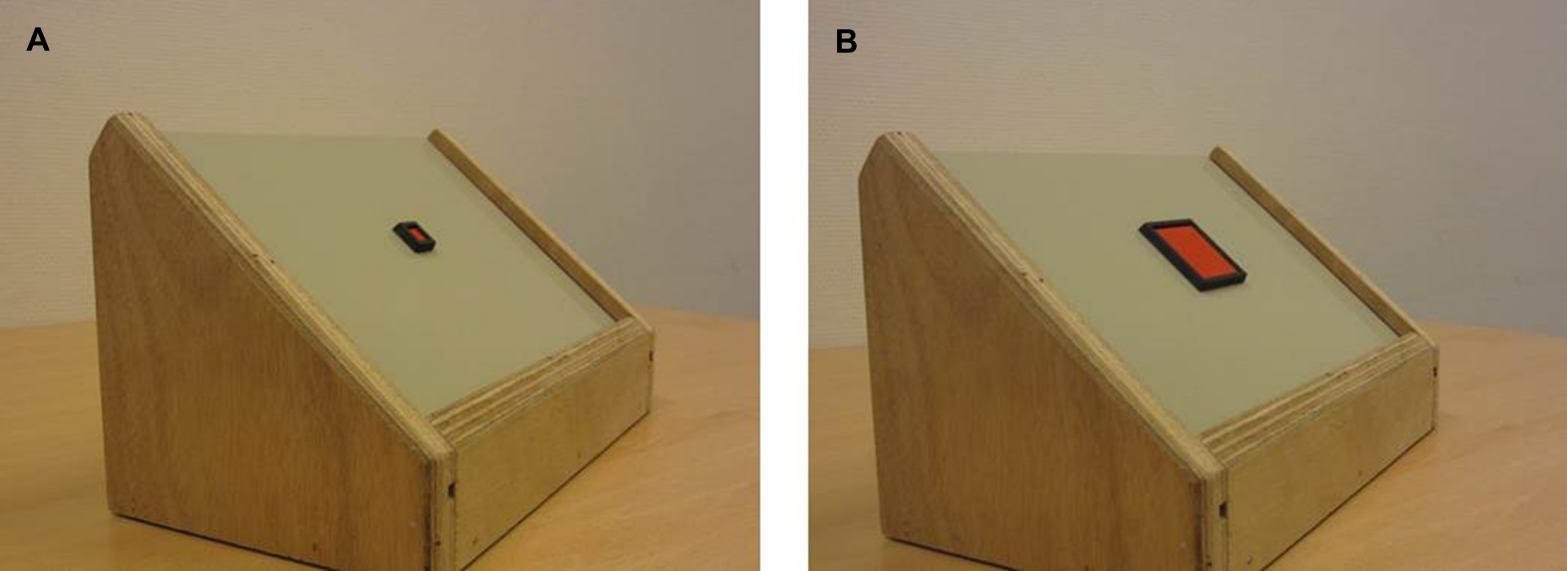
**The button press device.** The small button is presented at the left **(A)**, and the large button at the right **(B)**.

To assess the infants’ proficiency of aiming at and pressing large and small buttons, a button press device was constructed (see **Figure 3**). The device consisted of a wooden frame, in which boards with a single, red button could be fitted. Two boards were used, one with a small (1 by 1 cm) button, and one with a large button (4 by 4 cm) in the middle of the board. As the initial starting position of a reaching infant’s hand is relatively difficult to control, manipulating distance was expected to be difficult. Therefore, only button size was manipulated in the execution task. To ensure that infants would aim precisely at the button instead of pushing it with their whole hand, the buttons were inlaid into the surface, with a black edge around them. Pressing elicited a sound to enhance infants’ motivation to try to succeed in pressing the button.

### Procedure

The procedure for data collection was kept as similar as possible across age groups. Participating infants were seated in a car chair resting on the lap of their caregiver in front of a computer monitor. Participating adults were seated on an office chair adjusted to their height. Infants’ gaze was recorded using a Tobii 1750 (Tobii Technology, Sweden). Adults’ gaze was recorded with a different, but comparable eye-tracker (Tobii T120; Tobii Technology, Sweden), as adults were tested for a different, unrelated study at the same occasion. All participants first underwent a calibration procedure in which a contracting and expanding circle accompanied by a sound was shown on nine locations on the screen, forming a 3-by-3 grid. The calibration was accepted, if data was available for seven or more calibration points. The calibration procedure took between 2 and 5 min time. Immediately after calibration, the experiment started, which consisted of 96 (adults) or 48 (infants) trials. Trials were presented in pseudo-random order and were interleaved with brief attractive audiovisual clips to maintain the attention of the participants to the screen (16 for the infants, 3 for the adults). Stimulus presentation took 7 min for the adults and between 3 and 4 min for the infants (some infants were very attentive and in these cases some of the attention getters could be omitted). Trials were randomized within blocks, such that each block consisted of a random sequence of all eight unique stimulus videos. Infants thus observed 6 blocks and adults 12 blocks.

After the eye-tracking experiment, infants who had been sitting in the car seat were put on their parents lap. They were presented with the button pressing device, which stood on the table in front of them. Their actions were recorded with a video camera (Sony handycam DCR-SR190, frame rate: 25 Hz). They were first asked to try to press the large button, then the small button, followed by again the large and then the small button. The large button was presented first to maximize the chances that infants would try out both buttons. Had first the small button been presented, some infants might have started with a failure, diminishing the chances that they would continue with the other button. Presenting the small button first might have caused a selective drop-out as the younger infants were expected to have problems pressing the small button. The experimenter demonstrated how to press the button and encouraged the infant to follow her example in case infants were hesitant to press the button themselves. Infants were tested until they lost interest or for maximally 1.5 min per button type (large or small). On average, infants explored the large button for 56 s in case of the large button, and 55 s in case of the small button. One 15-month-old did not attempt pressing any of the buttons. In addition, one 9-month-old and two 15-month-olds did not show clear attempts pressing the small button but did demonstrate attempts pressing the large button (see **Table 1**).

**Table 1:** Minimum, maximum, SD, and average number of valid attempts to aim and press the button per age group and button size.

Age group	Button size	Minimum	Mean (SD)	Maximum
9-month-olds	Large	3	12 (7)	27
	Small	1	9 (7)	25
12-month-olds	Large	2	8 (5)	21
	Small	0	5 (3)	9
15-month-olds	Large	0	9 (6)	21
	Small	0	6 (4)	20

The eye-tracking task always preceded the button press task as infants tend to become restless over time during a testing session, and the button press task allowed for more movement of the infant than the eye-tracking task. Previous research has shown that only motor training but not observational training affects later perception of the trained action (Gerson and Woodward, 2014a,b; Gerson et al., 2015), and therefore no carry-over effects were expected.

### Gaze Data Analyses

Square-shaped areas of interest (AoIs) of equal size (100 by 100 pixels) were defined around the buttons in the stimulus displays, and in addition, an AoI was defined containing the full display of the stimulus movie (1280 by 580 pixels). First, the stimuli that were attended to were counted per participant and per condition. A stimulus was considered “watched” if at least one fixation fell on the full stimulus AoI while the stimulus video was playing. Second, per condition, trials were counted in which the participants fixated at one of the two button AoIs after onset of the hand movement and before the hand reached the AoI of the close button. These target fixations are subsequently referred to as “anticipatory looks.” A percentage of trials in which participants showed an anticipatory look to one of the buttons was calculated based on the total number of watched trials in that condition. In trials in which participants looked at both buttons during the anticipation interval, the trial would count both as a target and a non-target anticipation. Repeated measures ANOVAs were used to investigate whether participants correctly predicted whether a button served as the target of the action or not.

### Video Coding of Button Presses

Infants’ attempts to press the large and small buttons were coded from the video-recordings. Per type of button, the attempts to press the button were counted. Behavior was considered as an attempt to press the button if the infant’s hand touched the board in which the button was embedded while the infant looked at the button. Button press attempts were considered successful if the infant touched the button while looking at it. Attempts in which the infant was being moved or helped by their caregiver were excluded from the analyses. Beside success on the task, we were interested in the quality of the infant’s aiming. A well-aimed button press needs no correction in the movements, such that the aiming hand or finger lands directly on the button instead of first on the surroundings of the button. Movement correction was quantified as the time between the first moment the device was touched and the first moment the button was touched. Accurate initial aims would result in short (down to 0 s) movement correction times. If an infant had no successful button press attempts for one of the buttons, no data was available for the movement correction time of that button.

## Results

### Action Perception

**FIGURE 4:**
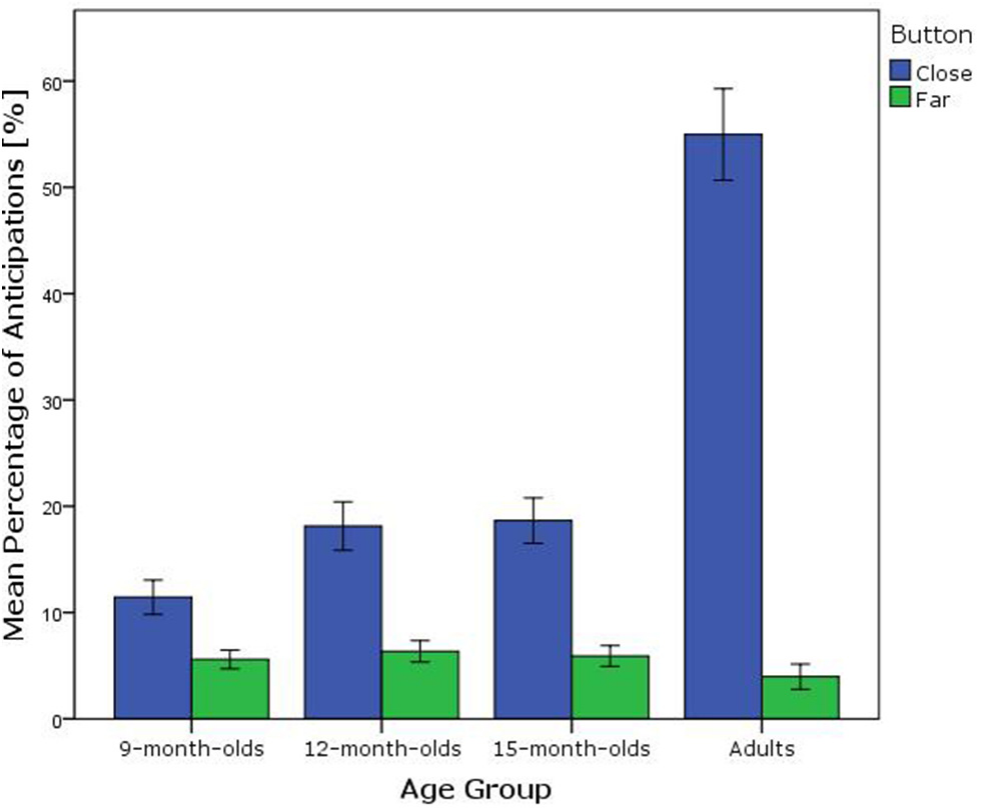
**Mean percentage of visual anticipations to the button close or far from the initial position of the hand, regardless of condition, split by age group.** Error bars represent 1 SEM.

The action in the stimulus display became disambiguated once the hand reached the close button, as then either the hand stayed on the close button, or continued to the far button. Thus, importantly, only anticipatory fixations initiated during this first ambiguous phase of the action were analyzed (the duration of the ambiguous phase ranged from 1.58 to 1.88 s after stimulus onset). An implication of this analysis choice was that fixations to the close button would likely occur more frequently compared to fixations to the far button, because for the latter, gaze needed to be more ahead of the action in space and time to reach the button during that period. Inspection of the data substantiated this assumption. **Figure 4** and **Table 2** display the mean percentage of fixations to the close button (closest to the initial position of the hand) and the far button (further from the initial position of the hand) during the analysis window collapsed over conditions. Given that participants tended to anticipate only to the close button and appeared to exhibit hardly any anticipations to the far button, the subsequent conditional analyses will focus on anticipations to the close button, which was either the target of the action, or not.

**Table 2:** Minimum, maximum, SD, and average number of observed trials per age group and button location.

Age group	Button location	Minimum	Mean (SD)	Maximum
9-month-olds	Close	9	20.1 (4.5)	24
	Far	7	19.4 (5.0)	24
12-month-olds	Close	7	20.1 (4.8)	24
	Far	10	20.8 (4.4)	24
15-month-olds	Close	17	22.3 (1.8)	24
	Far	18	22.7 (1.5)	24
Adults	Close	40	47.6 (1.9)	48
	Far	40	47.6 (1.9)	48

A repeated measures ANOVA was conducted to analyze the frequency of anticipatory looks to the close button with button function as a within-subjects factor (target, non-target) and age group (9-month-olds, 12-month-olds, 15-month-olds, adults) as a between-subjects factor. There was a main effect of age on the percentage of anticipatory looks [*F*(3,97) = 50.33, *p* < 0.001, $\eta_{p}^{2}$ = 0.61]. *Post hoc* independent samples *t*-tests showed that adults displayed a higher percentage of anticipatory looks (*M* = 55%, SD = 18) than the 15-month-olds [*M* = 19%, SD = 11, *t*(25.4) = 7.55, *p* < 0.001]^1^ and the 12-month-olds [*M* = 18%, SD = 12, *t*(26.5) = 7.56, *p* < 0.001]. No difference was found in anticipatory looks between the 15- and 12-month-olds [*t*(54) = 0.17, *p* = 0.867]. The 9-month-olds showed less frequent anticipatory looks (*M* = 11%, SD = 8) than the 12- [*t*(53) = 2.38, *p* = 0.021] and 15-month-olds [*t*(53) = 2.68, *p* = 0.010].

**FIGURE 5:**
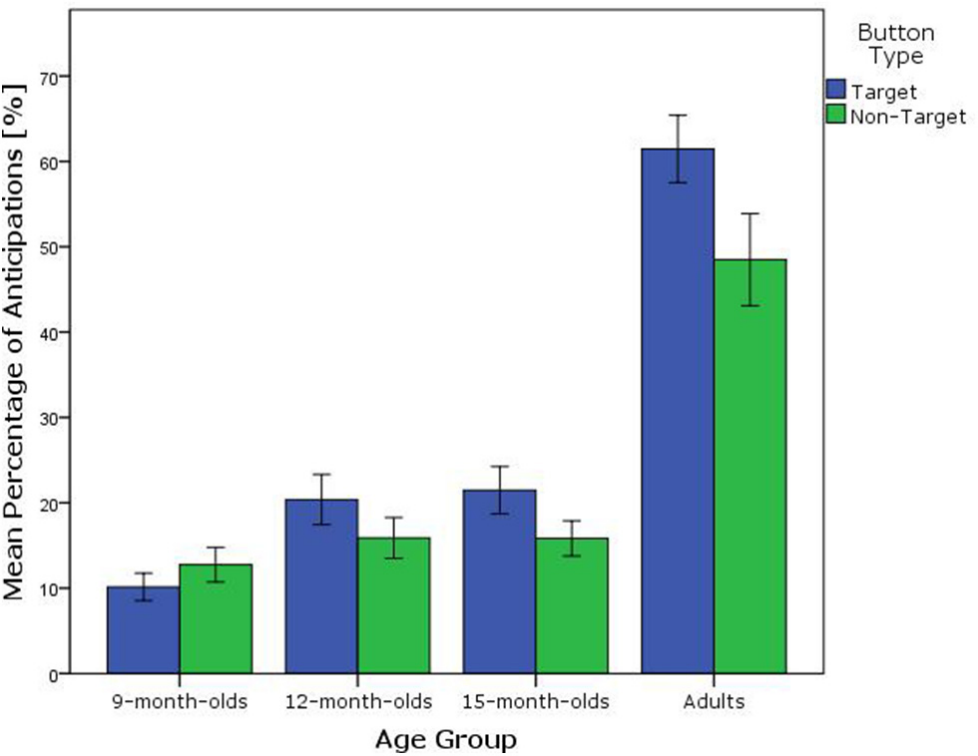
**Percentage of anticipatory looks to the close button when it was the target (blue bars) or not (green bars) split by age group.** Error bars represent 1 SEM.

A main effect of button function was observed [*F*(1,97) = 14.56, *p* < 0.001, $\eta_{p}^{2}$ = 0.13], indicating that across age groups, participants anticipated more frequently to the close button when it was the target (*M* = 25%; SD = 22) compared to when it was not the target button (*M* = 21%; SD = 19). A significant interaction effect was found [*F*(3,97) = 5.09, *p* = 0.003, $\eta_{p}^{2}$ = 0.14], indicating that the age groups differed in the frequency of anticipatory looks to the target compared to the non-target button. To further verify that the interaction effect was not solely due to the difference between adult and infant performance, an ANOVA was run without the adult data. A marginally significant main effect of button function was found [*F*(1,80) = 3.38, *p* = 0.070, $\eta_{p}^{2}$ = 0.04], together with a significant interaction effect of age group and button function [*F*(2,80) = 3.51, *p* = 0.035, $\eta_{p}^{2}$ = 0.08]. Planned paired comparisons for the separate age groups revealed that adults anticipated more frequently to the button when it was the target compared to when it was not [*t*(17) = 3.32, *p* = 0.004]. The same was the case for the 15-month-olds [*t*(27) = 2.37, *p* = 0.025], whereas the 12- and 9-month-olds did not look more frequently at the close button when it was the target compared to when it was not [12-month-olds: *t*(27) = 1.59, *p* = 0.125, 9-month-olds: *t*(26) = -1.45, *p* = 0.141; see **Figure 5** and **Table 3**].

**Table 3:** Minimum, maximum, SD, and average frequency of anticipation (%) per age group and button function.

Age group	Button function	Minimum	Mean (SD)	Maximum
9-month-olds	Target	0	10.2 (8.3)	33.3
	Non-target	0	12.8 (10.6)	36.1
12-month-olds	Target	0	20.4 (15.6)	62.5
	Non-target	0	15.9 (12.7)	41.7
15-month-olds	Target	0	21.5 (14.7)	56.0
	Non-target	0	15.8 (10.9)	37.5
Adults	Target	37.5	61.5 (16.8)	89.6
	Non-Target	12.5	48.5 (22.9)	87.5

### Action Production

A repeated measures ANOVA was used to analyze the effect of button size (small, large) and age group (9-month-olds, 12-month-olds, 15-month-olds) on the percentage of successful button press attempts out of all attempts. A main effect of button size on the percentage of successful button presses was found [*F*(1,68) = 28.05, *p* < 0.001, $\eta_{p}^{2}$ = 0.29], indicating that the infants were more successful in pressing the large (*M*_large_ = 88%, SD_large_ = 22) compared to the small button (*M*_small_ = 69%, SD_small_ = 37). Furthermore, the interaction between age group and button size was found to be significant [*F*(2,68) = 15.18, *p* < 0.001, $\eta_{p}^{2}$ = 0.31]. Independent samples *t*-tests showed that the 12-month-olds were more successful than the 9-month-olds when trying to press the small button [*t*(32.9) = 5.79, *p* < 0.001], but no significant differences were found between these groups when trying to press the large button [*t*(42) = 0.51, *p* = 0.611]. In addition, the percentage of successful button presses was found to depend on age [*F*(2,68) = 15.18, *p* < 0.001, $\eta_{p}^{2}$ = 0.31], with the 12-month-olds showing more successful button presses than the 9-month-olds [9-month-olds: *M*_small_ = 38%, SD_small_ = 34, *M*_large_ = 85%, SD_large_ = 12; *t*(42) = 4.73, *p* < 0.001]. The success rates of the 12-month-olds for the small and large button (*M*_small_ = 86%, SD_small_ = 19, *M*_large_ = 88%, SD_large_ = 24) were not different from the 15-month-olds [*M*_small_ = 81%, SD_small_ = 35, *t*(47) = 0.65, *p* = 0.522; *M*_large_ = 90%, SD_large_ = 26, *t*(48) = -0.29, *p* = 0.771].

An identical repeated measures ANOVA was conducted on the movement correction time data. A main effect of button size was observed [*F*(1,63) = 53.81, *p* < 0.001, $\eta_{p}^{2}$ = 0.46], as significantly more time was needed to correct the aiming movement to a small (*M*_small_ = 0.52 s, SD_small_ = 0.54) than to a large button (*M*_large_ = 0.08 s, SD_large_ = 0.14). The interaction between age group and button size had a significant effect on the movement correction times [*F*(2,63) = 6.69, *p* = 0.002, $\eta_{p}^{2}$ = 0.18]. The three age groups were equally fast in pressing the large button (*M*_9__months_ = 0.09 s, SD_9__months_ = 0.12, *M*_12__months_ = 0.10, SD_12__months_ = 0.12, *M*_15__months_ = 0.06, SD_15__months_ = 0.18, all *t*s < 1.0, all *p*s > 0.308). However, the 15-month-olds needed less time for correcting their movements than the other two groups when aiming for the small button (*M*_9__months_ = 0.82 s, SD_9__months_ = 0.79, *M*_12__months_ = 0.50 s, SD_12__months_ = 0.32, *M*_15__months_ = 0.27 s, SD_15__months_ = 0.15; *t*s > 3.0, *p*s ≤ 0.006), whereas the 9- and 12-month-olds differed only marginally in this respect [*t*(26.8) = 1.71, *p* = 0.099]. Furthermore, movement correction time was dependent on age [*F*(2,63) = 6.93, *p* = 0.002, $\eta_{p}^{2}$ = 0.18], which was caused by differences in aiming for the small button.

### Learning Effects

The set of video stimuli consisted of eight unique movies which were repeated six times for the infants and 12 times for the adults. Potentially, the found effects might hence be due to learning during the experiment. To investigate whether learning had occurred, the average anticipation frequency was calculated per block, per individual and split by condition. The anticipation frequencies were subjected to a six (blocks) by two (button function) by four (age group) mixed ANOVA. There are two results of relevance for the question of learning effects. First, an interaction between block and button function could indicate learning throughout the age groups. This interaction was found to be not significant [*F*(5,420) = 1.09, *p* = 0.364]. The second relevant result is the three-way interaction between block, button function, and age group. A significant interaction might indicate that the younger two groups did not show learning within the experiment whereas the other two groups did display learning effects. This three-way interaction was found to be marginally significant [*F*(15,420) = 1.57, *p* = 0.078]. To verify whether this indeed indicates that the older two age groups learnt when the close button was the target and when not, a follow-up six by two by two ANOVA was conducted only including the data of the 15-month-olds and the adults. If learning is to explain the differences found between the younger two groups and the older groups, then this ANOVA should yield a significant interaction between block and button function. This interaction was not found to be significant [*F*(5,215) = 0.64, *p* = 0.673], which shows that learning during the experiment cannot explain the differences found in predictions between the 9- and 12-month-olds on the one hand, and the 15-month-olds and adults on the other hand. More details on the analyses of potential learning effects can be found in the supplementary materials.

### Relation between Action Observation and Action Production

The results presented above show that success rates in aiming at the small button improved between 9 and 12 months of age and movement correction times decreased between 12 and 15 months of age. The ability to make velocity-based predictions develops in parallel, as 15-month-olds displayed velocity-based predictions, whereas 9- and 12-month-olds did not. To study the relation between action observation and action performance more closely, we examined the group of 12-month-olds, as this was the transitional group consisting of infants who were at the verge of learning to use velocity of natural movements to predict actions. A correlation analysis was performed to investigate whether action production and action prediction skills were related at the level of the individual infants. In the correlation analyses, proficiency in aiming at the small button was used as the measure of interest, as this reflects the ability to aim with high precision best. The time needed to correct the aiming movements to the small button was not found to be related to the prediction accuracy, expressed as the difference between the percentage of target and non-target anticipations (*p* = 0.654, controlling for age in days). Likewise, the relation between the success rate of aiming at the small button was not found to be related with action prediction accuracy (*p* = 0.902, controlling for age in days).

## Discussion

The aim of the current study was to examine whether the velocity of a natural movement, as manipulated through manipulating the size of and the distance to the targets, is used by an observer to predict whether an object will be the target of the observed action, and if so, whether motor development and hence the motor system is crucial for these predictions to emerge. Gaze data showed that adults and 15-month-old infants more frequently displayed visual anticipations to a button when it was the target compared to when it was not. No learning over trials was observed. The speed-accuracy trade-off, slower movements toward smaller targets, and the two-thirds power law expressing a related velocity dependent phenomenon, namely slower movements allow higher bell-shaped movement trajectories (Lacquaniti et al., 1983), are the only lawful relations that can have been acquired prior to the study. The results thus indicate that 15-month-olds and adults based their predictions on the speed of an observed movement, as velocity was the central cue for distinguishing targets from non-targets. In contrast, infants of 9 and 12 months of age did not show any indications that they used the speed information of the observed movement for their action predictions. This was congruent with the development of producing this action: 15-month-olds were more proficient in aiming at and pressing a button accurately than the 12- and 9-month-olds. This suggests that the motor system underlies velocity-based predictions.

Three factors influenced how frequently the observers looked at the buttons while the action was unfolding. First, many more anticipatory looks were made to the button nearest to the initial position of the hand than to the button located further away, when the hand had not yet passed the nearest button. However, our analysis period ended when the hand was at the point of passing the nearest button, because once the hand had passed the close button, it was obvious that the far button was the target. As a consequence, to be counted as a predictive look, observers had to be more ahead of the action when predicting the far button than when predicting the close button. Due to the low base rate of predictions to the far button, only the predictions to the close button could be analyzed. Future studies could overcome this distance problem by using 3D environments such as virtual reality to, for instance, create an ambiguous situation in which the targets create an equally sized image on the retina but differ in distance to the observer. The speed of the movement might then disambiguate the situation.

The second factor that influenced anticipatory looks was the velocity of the natural movement, which was the main factor in the current study which was manipulated by means of using differently sized targets placed at two distances. The results showed that participants looked more frequently at the close button when it was the target compared to when it was not, which indicates that the participants made use of the velocity information of the hand to predict which button would be pressed.

The third factor that affected the frequency of anticipatory looks was age. Whereas adults and 15-month-old infants looked more frequently to the close button when it was the target compared to when it was not, 9- and 12-month-old infants did not show this difference.

Velocity-based predictions may result from action simulation in the motor system of the observer. The motor system has been shown to respond stronger to the observation of actions that have to be performed with more accuracy (Eskenazi et al., 2011). The speed people expect to see during an observed action matches the actual speed of the performed action (Grosjean et al., 2007; Eskenazi et al., 2009), which illustrates that the action-perception link also plays a role in the speed-accuracy trade-off (cf. Rizzolatti et al., 1996; Hari et al., 1998; Flanagan and Johansson, 2003; Cattaneo et al., 2007). However, thus far, observation of the speed-accuracy trade-off has primarily been studied in adults, which left the question unanswered how the perception of the speed-accuracy trade-off develops. Given these prior findings, the hypothesis of the current study was that the motor system not only underlies *post hoc* judgments of the observed velocity of movements, but also facilitates on-line predictions made while the action still unfolds. Our results are in line with this hypothesis: the action prediction performance of the 15-month-old infants suggested that they use velocity information in action prediction, whereas the 9- and 12-month-olds seemed not to integrate the observed velocity in their predictions of the observed actions. The tested 15-month-old infants were also better at pressing buttons than the 9- and 12-month-olds. Using velocity information to predict which button will be pressed thus follows – at least by and large – the same developmental time course as the ability to press buttons. This is in line with previous infant research showing that motor ability affects action perception (van Elk et al., 2008; Kanakogi and Itakura, 2011; Ambrosini et al., 2013; Gerson and Woodward, 2014a,b; Gerson et al., 2015). However, within the group of 12-month-old infants, the individual button pressing proficiency was not found to be related to the ability to use speed for action prediction. It might well be that our motor measurement was not sensitive enough to correlate motor performance with action prediction performance at an individual level. Nevertheless, it is interesting that the differences in motor performance at the group level overlap with the anticipatory eye capacities in the observation task. However, at least two alternatives can be given for the suggested improvement in terms of motor simulation. First, visual experience acquired between 12 and 15 months of age may contribute to velocity-based predictions as well (Hunnius and Bekkering, 2014). Second, the effects observed could also be related to a general maturation pattern of the brain that enables both action execution as well as action observation. The importance of visual experience and brain maturation in the development of velocity-based predictions can be tested in future research by investigating whether 15-month-olds can use velocity information for the prediction of actions that are not yet part of their motor repertoire. Furthermore, it would be interesting to study groups with delays in motor development to gain more knowledge about whether or not motor experience is necessary for velocity-based predictions.

## Conclusion

We found empirical evidence that observers can predict whether an object will be the target of an action based on the velocity of the observed natural movement, which was manipulated through manipulating the size of and the distance to the target objects. In the current study, the action target was a button. Fifteen-month-old, but not 9- and 12-month-old infants showed an adult-like prediction pattern, suggesting that at 15 months of age, infants are beginning to use velocity to inform their predictions of other’s button pressing actions. The 15-month-olds were more proficient in performing this type of action compared to the 9- and 12-month-olds. Together, this indicates that the development of velocity-based predictions follows a time line corresponding to the development of motor skill of the predicted action. Future research should parse out the roles of visual and motor experience for action prediction. Being a proficient actor may turn out to be necessary in order to accurately predict what other people are planning to do.

## Conflict of Interest Statement

The authors declare that the research was conducted in the absence of any commercial or financial relationships that could be construed as a potential conflict of interest.

## Footnotes

1 In case equal variances could not be assumed as indicated by a significant outcome of Levene’s test for equality of variances, adjusted dfs are reported.
