# Supplementary material for: Fifteen-month-old infants use velocity information to predict others’ action targets
Source: Front Psychol. 2015 Aug 4;6:1092. doi: 10.3389/fpsyg.2015.01092 (PMC4523741; doi:10.3389/fpsyg.2015.01092)
Supplement: Supplementary file 1 [file Data_Sheet_1.PDF]

Supplementary materials

Learning effects

As described in the main article, participants anticipated the target button more frequently than the non-target button. An interaction with age group was found, indicating that the older two age groups (15-month-olds and adults) indeed anticipated the target button more often than the non-target, while this difference was not found in the two younger age groups (9- and 12-month-olds). During the experiment, 8 unique stimulus videos were repeatedly displayed, leading to 6 repetitions of each video for the infants, and 12 repetitions for the adults. In each stimulus video, goal-attainment was shown, which might have let the older age groups to learn during the experiment that certain low-level perceptual features were associated with certain outcomes. To examine whether a low-level learning effect might explain the results, several ANOVAs were conducted. In the first ANOVA, all age groups were included. The second ANOVA zoomed in on the older two age groups. For the ANOVAs, the frequencies of anticipation to the target and to the non-target button were averaged over each block of 8 trials. For the sake of comparison, only the first 6 blocks in all age groups were considered (adults observed 12 blocks in total).

A 2 by 6 by 4 mixed ANOVA was conducted with button function (target, non-target) and block (block 1, block 2, block 3, block 4, block 5, block 6) as within-subjects factors and age group (9-, 12-, 15-month-olds, adults) as a between-subjects factor. A main effect of button function was found ($F(1,84) = 15.47$, $p < 0.001$), indicating that the target button was more frequently anticipated (M = 24%, SD = 22) than the non-target button (M = 20%, SD = 19). Furthermore, button function and age group were found to have a marginally significant interaction effect on the anticipation frequency ($F(3, 84) = 2.11$, $p = 0.105$). Post-hoc paired samples t-tests display the same pattern as in the main analyses described in the article: adults and 15-month-olds anticipated the target (M_{adults} = 59%, SD_{adults} = 18, M_{15mo} = 21%, SD_{15mo} = 15) more frequently than the non-target (M_{adults} = 50%, SD_{adults} = 22, M_{15mo} = 14%, SD_{15mo} = 10; adults: $t(17) = 2.34$, $p = .032$; 15-month-olds: $t(27) = 2.85$, $p = .008$) whereas the other two age groups did not show this discrimination (target: M_{9mo} = 11%, SD_{9mo} = 8, M_{12mo} = 20%, SD = 16; non-target: M_{9mo} = 12%, SD_{9mo} = 12, M_{12mo} = 15%, SD = 12; 9-month-olds: $t(26) = -0.70$, $p = .490$; 12-month-olds: $t(27) = 1.70$, $p = .102$). In addition, a main effect of block was found ($F(5,420) = 5.10$, $p < .001$). Paired-samples t-tests analyzing the differences between consecutive blocks indicate that the frequency of anticipations increased between block 1 and 2 (M_{block1} = 19%, SD_{block1} = 20, M_{block2} = 25%, SD_{block2} = 24; $t(99) = -3.25$, $p = .002$), stays stable over blocks 2, 3, 4 and 5 (ts < 1.38 and ps > .173), and decreases between block 5 and 6 (M_{block5} = 25%, SD_{block5} = 24, M_{block6} = 19%, SD_{block6} = 22; t(89) = 3.48, $p = .001$). However, the interaction between button function and block was not found to be significant ($F(5,420) = 1.09$, $p = .364$), which means that the conditional differences were in general stable throughout the experiment. The effect of block was not modulated by age as the interaction term was not significant ($F(15,420) = 1.19$, $p = .277$). Most crucial for the current analysis however was the three-way interaction between button function, block and age group. This interaction was found to be marginally significant ($F(15, 420) = 1.57$, $p = .078$). This might indicate that the older two age groups differentiated between the target and non-target

through learning whilst the other two age groups did not display such learning. To verify whether this potential explanation holds, a second ANOVA was performed.

A 2 by 6 by 2 mixed ANOVA was employed with button type and block as within-subjects manipulations and age group as a between-subjects manipulation. The results largely overlap with the results of the first ANOVA: A main effect of button function ($F(1,43) = 12.85$, $p = .001$) was found, together with a significant effect of block ($F(5,215) = 4.25$, $p = .001$). The interaction between button function and age group was not found to be significant ($F(1,43) = 0.15$, $p = .702$) indicating that the 15-month-olds reached adult-levels of functioning. Age group and block were not found to interact ($F(5,215) = 1.55$, $p = .176$). Crucial for the current analysis was whether the conditional differences increased with increasing block number. This appeared not the be the case as block and button function did not have an interaction effect on the frequency of anticipatory looks ($F(5, 215) = 0.64$, $p = .67$). The three-way interaction between button function, block and age group was marginally significant ($F(5,215) = 1.87$, $p = .102$). To further investigate the nature of this three-way interaction, separate repeated measures ANOVAs were run on the data of the two older age groups, with button function and block as within-subjects factors. In the ANOVA of the 15-month-olds, a main effect of button function ($F(1,26) = 7.34$, $p = .012$) and main effect of block was found ($F(5,130) = 2.89$, $p = .017$), but the interaction between block and button type was not found to be significant ($F(5,130) = 0.91$, $p = .479$). The ANOVA of the adult data yielded a similar pattern of results, with a significant main effect of button function ($F(1,17) = 5.48$, $p = .032$) and a significant main effect of block ($F(5,85) = 2.54$, $p = .035$). The interaction between button function and block was not significant ($F(5,85) = 1.17$, $p = .329$). Hence, the three-way interactions found in the previous ANOVAs including multiple age groups was not a result of learning effects in the older age groups. To verify whether the adult sample did not show learning during the experiment, an additional repeated measures ANOVA was conducted with button function and block number as within-subjects factors, including all 12 blocks. As expected, button function had a significant impact on anticipation frequency ($F(1,17) = 10.32$, $p = .005$). No main effect of block number was observed ($F(11,187) = 1.09$, $p = .374$), nor an interaction effect of block number and button function ($F(11,187) = 1.06$, $p = .395$), indicating that there were no significant learning effects in the later blocks either. In conclusion, the ANOVAs conducted investigating whether potential learning effects can explain the data revealed that learning can be ruled out as an explanation of the conditional differences.
